# Editorial: Mathematical and statistical modeling of infection and transmission dynamics of viral diseases

**DOI:** 10.3389/fpubh.2023.1295976

**Published:** 2023-10-06

**Authors:** Kayode Oshinubi, Pierre Magal, Olumide Longe, Jacques Demongeot

**Affiliations:** ^1^School of Informatics, Computing and Cyber Systems, Northern Arizona University, Flagstaff, AZ, United States; ^2^Individual Based Modeling, UMR CNRS 5251, University Bordeaux, Talence, France; ^3^Faculty of Computational Sciences and Informatics, Academic City University, Accra, Ghana; ^4^Laboratory AGEIS EA 7407, Team Tools for e-Gnosis Medical, Faculty of Medicine, University Grenoble Alpes (UGA), La Tronche, France

**Keywords:** viral epidemic, COVID-19, reproduction number, vaccination, modeling

## Introductory remarks on viral disease dynamics

The articles in this Research Topic show, many advances have been made in public health research, especially in the field of data collection, modeling (deterministic and random), and prediction: (i) public databases on COVID-19 have multiplied [see COVID-19 Open Data Repository (1) for example 301 public sites around the world allowing access to COVID-19 data], (ii) review articles on COVID-19 outbreak including statistics and models are numerous (in Frontiers in Public health alone, the site in WHO COVID-19 Research Database (2) counts 173 articles under heading Systematic review/Meta-Analysis) and (iii) the number of articles using a formal approach predicting COVID-19 waves is constantly growing (Google Scholar lists for example 144,000 articles with the query “modeling and forecasting COVID-19 pandemic”).

The article in this Research Topic is useful for the modeling of viral disease dynamics.

## The various facets of the recent mathematical and statistical models of viral disease data in this Research Topic

In the Research Topic entitled “*Mathematical and statistical modeling of infection and transmission dynamics of viral diseases*,” the twelve papers are representative of different mathematical and statistical approaches to the recent COVID-19 pandemic, Hepatitis A/B virus, and Mpox disease data:

We first present articles that deals with the recent COVID-19 pandemic. In Tormos et al. analyzed the role of in-person school reopening in Spain on the evolution of COVID-19 infections using an interrupted time-series perspective by exploring the dataset and modeling the dynamics of the disease in different Spanish regions and autonomous communities that reopened schools at varying moments in time during September 2020. Their analysis suggests that school reopening may generate a retro-feedback of the disease spread with parents' return to work and social activity, leading to exponential growth, as observed in Catalonia and other Spanish autonomous communities during September and October of 2020. Naffeti et al. established that the geography of the COVID-19 pandemic in Africa largely overlaps with the geography of the wealth of the 30 countries considered by using spatiotemporal evolution techniques, which take into consideration demographic, economic, and environmental aspects that can better explain the geographical variations of the basic reproduction rate at the early beginning of each wave of the pandemic. BuHamra et al. used self-developed natural language processing to automate the extraction of causes of death and comorbidities from the electronic health records of COVID-19 outbreak from the start of the pandemic until the end of all major epidemic waves. The research findings proposed that to reduce misspellings or incorrect forms, organizing the electronic health record with well-defined sections and giving menu-driven options for reporting causes of death and comorbidities. Majeed et al. aim to examine the impact of SARS-CoV-2 during an influenza season and quantify the effects of the two respiratory infections co-circulating using a mathematical model. The research investigates the best techniques to delay and split the peaks of the influenza outbreak and the COVID-19 wave among numerous scenarios and interventions. This study demonstrates that effectively managing and controlling both influenza and COVID-19 outbreaks during the same season depends on establishing optimal vaccine coverage techniques. KhudaBukhsh et al. investigated COVID-19 epidemic in Marion Correctional Institution in the spring of 2020 using a thorough and statistically sound technique. The analysis is based on a compartmental mathematical transmission model that is fitted to data using dynamical survival analysis, which permits the computation of explicit likelihoods to summarize uncertainty. The research findings underscore the tremendous potential for respiratory infection transmission in prisons, as well as the crucial need for improved infection monitoring and reporting in correctional facilities.

Chu et al. identify a random long-term pattern of biweekly global new COVID-19 cases with a seasonal feature. Most countries have co-integration linkages of newly reported instances of distinct varieties of concerns, regardless of their demographics or responses to the virus. The findings suggested that consistent techniques may be used to limit the spread. Furthermore, drastic eradication attempts may be ineffective, and there is a substantial risk that the COVID-19 pandemic may become an endemic. Janko et al. developed a framework to assist policymakers in developing plausible intervention tactics by dynamically changing non-pharmaceutical interventions using artificial intelligence to forecast the infection trends, aggregated the socioeconomic costs from the literature and expert knowledge, and used a multi-objective optimization algorithm to find and evaluate various non-pharmaceutical intervention plans. The model generates efficient intervention plans to fight a pandemic and can evaluate their effect and costs.

Furthermore, we present articles in this research theme that deals with Hepatitis A/B virus modeling. In Sun et al. work focuses on a fractional-order differential equation model with time delay and logistic proliferation to better understand the transmission mechanism of the Hepatitis B virus in the human body. Their research findings revealed that immune response time delay and fractional order can substantially impact the dynamic behavior of the Hepatitis B virus infection transmission. As a result, while modeling and investigating Hepatitis B virus infection, temporal delay and fractional order should be considered. Jeong et al. used a flexible spatio-temporal model to analyze the spatio-temporal fluctuations of the hepatitis A virus in Korea and the influence of socioeconomic and weather-related parameters. To evaluate the effects of risk factors, the authors developed a Bayesian spatiotemporal zero-inflated Poisson regression model of weekly hepatitis A virus incidence in Korea. This is the first study to build a spatiotemporal model of hepatitis A virus occurrence in Korea, considering numerous socioeconomic parameters. The proposed model will be beneficial in forecasting, preventing, and regulating the spread of the hepatitis A virus.

Mpox disease modeling is also considered by some research presented by authors in this Research Topic. Ngungu et al. provided a brief overview of the Mpox virus and its transmission dynamics by investigating its spread and the effect of a non-pharmaceutical intervention (quarantine). The work provides insight into the exponential growth rate of the Mpox virus dynamics prediction and how to stop it from spreading and understand the effects of non-pharmaceutical intervention on infected individuals, which will guide how to deploy intervention resources to contain the disease's spread. Yuan et al. developed a Susceptible-Exposed-Infected-Recovered (SEIR) modeling framework to evaluate the impact of vaccination and other disease control strategies. The vaccination of a high-risk group and ring vaccination strategy, as well as testing, isolating patients and contact tracing, are all explored, as are various interventions during gathering occasions. The authors primarily focused on evaluating the effectiveness of public health control methods, such as preventive vaccination or immunization post-exposure, to assist public health decision-making by simulating scenarios of gatherings with varying numbers of attendance and levels of intervention. The research findings indicated that reactive ring vaccination may not be sufficient in and of itself; however, if close contacts of cases can be identified, vaccinated, and isolated, an outbreak following a mass gathering event may be avoided.

Lastly, other infectious disease modeling is considered by N'konzi et al. modified the basic deterministic Susceptible-Exposed-Infected-Recovered (SEIR) model to account for the effect of disease control measures as well as the feedback loop between non-pharmaceutical interventions adherence and disease dynamics. The model is used to study the impact of temporal fluctuations in non-pharmaceutical intervention adherence levels on infectious disease dissemination. To capture the dynamics of the public level of adherence to non-pharmaceutical interventions, authors leverage on the simulation of disease dynamics and expand on the health belief model. The model implies that treatments aimed at enhancing non-pharmaceutical interventions adherence may be far more valuable than raising overall non-pharmaceutical interventions stringency.

## Concluding remarks

All the contributions to the Research Topic on “*Mathematical and statistical modeling of infection and transmission dynamics of viral diseases*” have a focus on developing mathematical and statistical models that are well suited for real data, which are gathered through a process filtered by modeling constraints. The idea is to obtain the optimal model with strong predictive power that will match the collected data and can forecast the future evolution of the observed disease trends while applying the most appropriate and scalable inquiry techniques.

## Author contributions

KO: Conceptualization, Supervision, Validation, Writing—original draft, Writing—review and editing. PM: Conceptualization, Validation, Writing—review and editing. OL: Conceptualization, Validation, Writing—review and editing. JD: Conceptualization, Supervision, Validation, Writing—original draft, Writing—review and editing.
